# Addressing cortex dysregulation in youth through brain health check coaching and prophylactic brain development

**DOI:** 10.36922/itps.1472

**Published:** 2024-04-30

**Authors:** Kenneth Blum, Eric R. Braverman, Mark S. Gold, Catherine A. Dennen, David Baron, Panayotis K. Thanos, Colin Hanna, Igor Elman, Marjorie C. Gondre-Lewis, J. Wesson Ashford, Andrew Newberg, Margaret A. Madigan, Nicole Jafari, Foojan Zeine, Keerthy Sunder, John Giordano, Debmayla Barh, Ashim Gupta, Paul Carney, Abdalla Bowirrat, Rajendra D. Badgaiyan

**Affiliations:** 1 Division of Addiction Research and Education, Center for Sports, Exercise and Global Mental Health, Western University of Health Sciences, Pomona, California, United States of America; 2 The Kenneth Blum Behavioral and Neurogenetic Institute LLC, Austin, Texas, United States of America; 3 Faculty of Education and Psychology, Institute of Psychology, Eötvös Loránd University Budapest, Budapest, Hungary; 4 Department of Molecular Biology and Adelson School of Medicine, Ariel University, Ariel, Israel; 5 Division of Personalized Medicine, Cross-Cultural Research and Educational Institute, San Clemente, California, United States of America; 6 Centre for Genomics and Applied Gene Technology, Institute of Integrative Omics and Applied Biotechnology, Purba Medinipur, West Bengal, India; 7 Division of Personalized Recovery Science, Transplicegen Therapeutics, Llc., Austin, Tx., United of States; 8 Department of Psychiatry, University of Vermont, Burlington, Vermont, United States of America; 9 Department of Psychiatry, Boonshoft School of Medicine, Wright State University, Dayton, Ohio, United States of America; 10 Division of Personalized Medicine, Ketamine Clinic of South Florida, Pompano Beach, Florida, United States of America; 11 Department of Psychiatry, Washington University School of Medicine, St. Louis, Missouri, United States of America; 12 Department of Family Medicine, Jefferson Health Northeast, Philadelphia, Pennsylvania, United States of America; 13 Department of Psychology and Behavioral Neuropharmacology and Neuroimaging Laboratory on Addictions, Research Institute on Addictions, University of Buffalo, Buffalo, New York, United States of America; 14 Cambridge Health Alliance, Harvard Medical School, Cambridge, Massachusetts, United States of America; 15 Department of Anatomy, Howard University School of Medicine, Washington, D.C., United States of America; 16 Department of Psychiatry and Behavioral Sciences, Stanford University, Palo Alto, California, United States of America; 17 Department of Integrative Medicine and Nutritional Sciences, Thomas Jefferson University and Hospital, Philadelphia, Pennsylvania, United States of America; 18 Department of Human Development, California State University at Long Beach, Long Beach, California, United States of America; 19 Awareness Integration Institute, San Clemente, California, United States of America; 20 Department of Health Science, California State University at Long Beach, Long Beach, California, United States of America; 21 Department of Psychiatry, University California, UC Riverside School of Medicine, Riverside, California, United States of America; 22 Future Biologics, Lawrenceville, Georgia, United States of America; 23 Division of Pediatric Neurology, University of Missouri Health Care-Columbia, Columbia, Missouri, United States of America; 24 Department of Psychiatry, Mt. Sinai School of Medicine, New York City, New York, United States of America

**Keywords:** Brain health check, Cognition, Dopaminergic dysregulation, Executive function, Reward deficiency syndrome, Genetics, Epigenetics

## Abstract

The Carter Center has estimated that the addiction crisis in the United States (US), if continues to worsen at the same rate, may cost the country approximately 16 trillion dollars by 2030. In recent years, the well-being of youth has been compromised by not only the coronavirus disease 2019 pandemic but also the alarming global opioid crisis, particularly in the US. Each year, deadly opioid drugs claim hundreds of thousands of lives, contributing to an ever-rising death toll. In addition, maternal usage of opioids and other drugs during pregnancy could compromise the neurodevelopment of children. A high rate of DNA polymorphic antecedents compounds the occurrence of epigenetic insults involving methylation of specific essential genes related to normal brain function. These genetic antecedent insults affect healthy DNA and mRNA transcription, leading to a loss of proteins required for normal brain development and function in youth. Myelination in the frontal cortex, a process known to extend until the late 20s, delays the development of proficient executive function and decision-making abilities. Understanding this delay in brain development, along with the presence of potential high-risk antecedent polymorphic variants or alleles and generational epigenetics, provides a clear rationale for embracing the Brain Research Commission’s suggestion to mimic fitness programs with an adaptable brain health check (BHC). Implementing the BHC within the educational systems in the US and other countries could serve as an effective initiative for proactive therapies aimed at reducing juvenile mental health problems and eventually criminal activities, addiction, and other behaviors associated with reward deficiency syndrome.

## 1. Introduction

The purpose of the brain health check (BHC) is to integrate objective assessments across cognition, neurological imaging, psychiatry, and genomics to identify youths who are at risk for juvenile mental health problems, criminal activities, addiction, and other behaviors associated with reward deficiency syndrome (RDS). Identifying vulnerable youths through these assessments can provide insights into proper interventions, such as genome-matched amino acid therapies that can treat reward/dopamine dysregulation and prevent the inheritance of epigenetic insults associated with addiction to future generations. Amidst the increasing drug abuse crisis in the United States (US) and the potential for long-term enormous societal costs, a brain research consortium developed this approach. The group is comprised experienced teachers, educators, drug abuse counselors, psychiatrists, clinicians, scientists, neuroscientists, geneticists, and addiction medicine physicians, who encourage the adoption of the standardized BHC in K1–K12 education. In addition, they endorse basic and clinical scientific research into brain health prophylaxis for developing brains.

## 2. Understanding reward dysregulation and potential therapeutic approaches

As defined in the Sage Encyclopedia of Psychiatric Disorders (2017), there is emerging evidence of an over-representation of the antecedent to RDS, encompassing both substance- and non-substance-related addictive behaviors, within the general US population.

It is well established that dopamine resistance in individuals with food and drug addiction is caused by dysfunctional genetic neurotransmitter polymorphisms, such as the A1 allele of the *DRD2* gene, and epigenetic insults. A burgeoning line of evidence shows that a natural, non-addictive, and safe putative D2 agonist may aid in the treatment of and recovery from these RDS behaviors in patients addicted to substances. The impact of the patented KB220 nutrigenomic technology, known as “Synaptamine Complex,” acts as an activator of the mesolimbic system, as observed through quantitative electroencephalography (qEEG) imaging. A published pilot study demonstrated that the intravenous administration of KB220 was observed to normalize the aberrant electrophysiological parameters of the reward circuitry site.^1^ The study also revealed that the qEEG graphs of an alcoholic and a heroin abuser with existing abnormalities (widespread theta and alpha activity, respectively) during protracted abstinence were significantly normalized after the administration of a single intravenous dose of KB220^®^ Synaptamine Complex Formulation.^1^ Both patients were genotyped for several neurotransmitter reward genes to determine if they carried any putative dopaminergic risk alleles that may predispose them to alcohol or heroin dependence, respectively. The genes examined included the dopamine transporter (*DAT1*, locus symbol *SLC6A3*), dopamine D4 receptor exon 3 *VNTR* (*DRD4*), *DRD2* TaqIA (rs1800497), *COMT* val158 met *SNP* (rs4680), monoamine oxidase A upstream *VNTR* (*MAOA-uVNTR*), and serotonin transporter-linked polymorphic region (*5HTTLPR*, locus symbol *SLC6A4*). It should be emphasized that these findings stem from case studies, and it is improbable for individuals to carry all putative risk alleles. Based on the previous research and our qEEG studies, we cautiously suggest that long-term activation of dopaminergic receptors may increase their proliferation, leading to enhanced “dopamine sensitivity” and a heightened sense of happiness, particularly in carriers of the *DRD2* A1 allele.^2^

The intravenous administration of the Synaptamine Complex Variant KB220 in >600 alcoholic patients resulted in a significant reduction in RDS behaviors; this effect was further supported by an expanded study involving oral KB220Z^3^ and functional magnetic resonance imaging conducted on abstinent heroin addicts.^4^ For a deeper understanding, future studies, including functional positron emission tomography scanning, are required to determine the acute and chronic effects of oral KB220Z on the number of D2 receptors and its interaction with the nucleus accumbens (NAc). In addition, further confirmation of these findings through large, population-based, and case-controlled experiments could ultimately lead to significant improvements in the treatment and recovery of patients with RDS and dopamine deficiency resulting from disruptions in the transduction of multiple neurotransmitter signals within the Brain Reward Cascade (BRC).^5^

**Figure 1.**
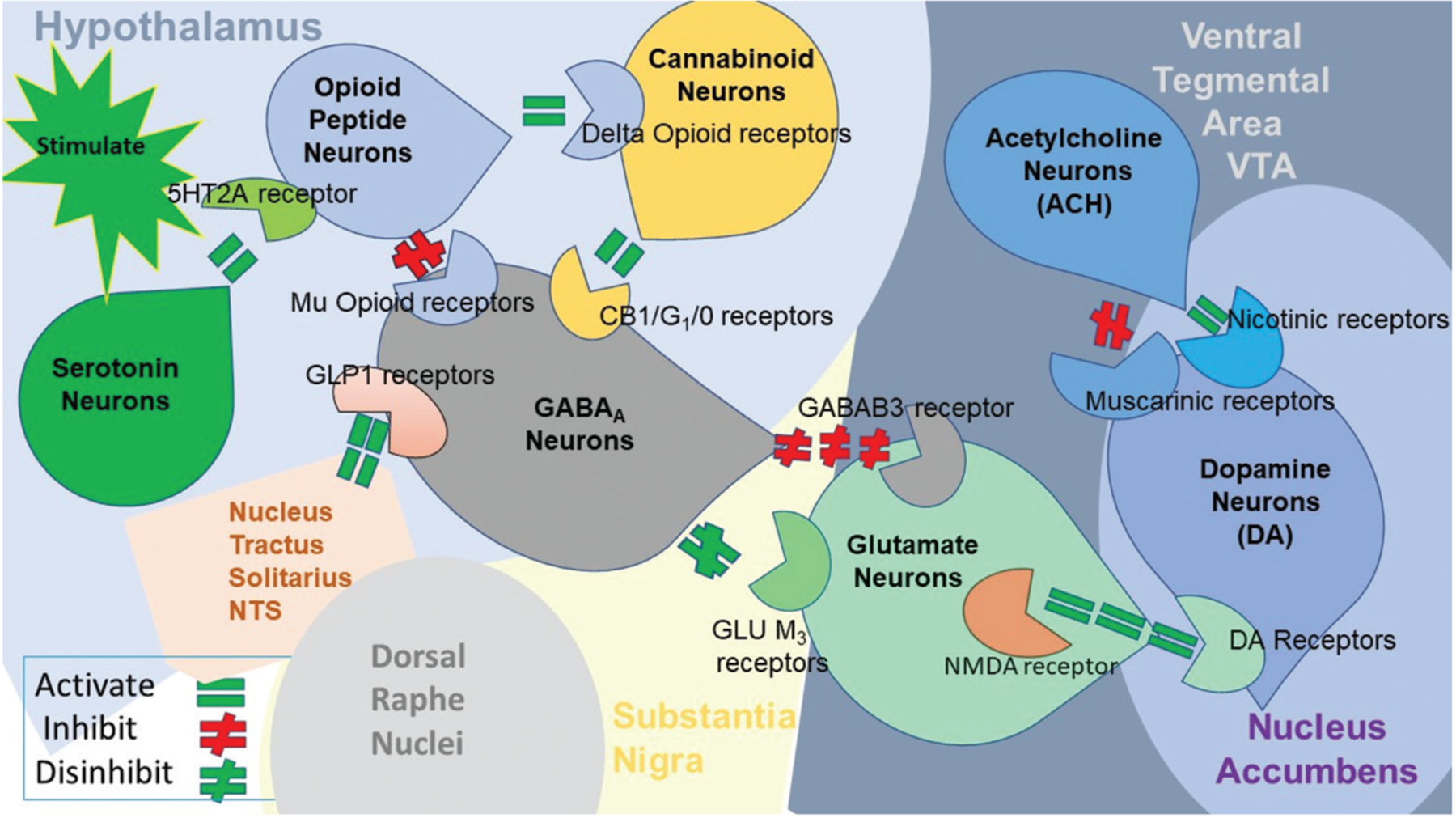
Interaction of at least eight major neurotransmitter-pathways involved in the brain reward cascade. In the hypothalamus, environmental stimulation triggers the release of serotonin, which, through receptors such as 5HT-2a, activates (green equal sign) the subsequent release of opioid peptides from opioid peptide neurons, also located in the hypothalamus. These opioid peptides, in turn, exert two distinct effects, possibly through two different opioid receptors. One effect inhibits (red hash sign) the mu-opioid receptor (possibly through enkephalin) and projects to GABAA neurons in the substantia nigra. The other effect stimulates (green equal sign) cannabinoid neurons (e.g., anandamide and 2-arachidonoylglycerol) through beta-endorphin-linked delta receptors, which further inhibit GABAA neurons in the substantia nigra. In addition, cannabinoids, primarily 2-arachidonoylglycerol, when activated, can indirectly disinhibit (red hash sign) GABAA neurons through the activation of G1/0 coupled to CB1 receptors in the substantia nigra. Not depicted in the figure, the dorsal raphe nuclei feature glutamate neurons that can indirectly disinhibit GABAA neurons in the substantia nigra through activation of GLU M3 receptors (red hash sign). When stimulated, GABAA neurons powerfully (red hash signs) inhibit VTA glutaminergic drive through GABAA neurons. It is also possible that stimulation of ACH neurons at the NAc can stimulate both muscarinic (red hash) and nicotinic (green hash) receptors. Glutamate neurons in the VTA project dopamine neurons through NMDA receptors (green equal sign) to preferentially release dopamine at the NAc, resulting in a sense of euphoria, or “wanting” response. Figure 1 also depicts that GLP1 from the nucleus tractus solitarius stimulates GABAA in the Substantia Nigra. As a result, dopamine release is low (endorphin deficiency), followed by feelings of unhappiness. On the other hand, overall (healthy) happiness depends on the optimal balance of dopamine, regulated by the dopamine homeostatic tonic set point.^6^

Moreover, recent neuroimaging studies have highlighted the potent effects of KB220Z, underscoring the importance of Pro-dopamine regulation along the BRC (Figure 1).

It is also possible that ACH neurons at the NAc ACH can stimulate both muscarinic (red hash) and nicotinic (green hash) receptors. Finally, glutamate neurons in the VTA will project to dopamine neurons through NMDA receptors (green equal sign) to preferentially release dopamine at the NAc (shown as a bullseye), indicating euphoria or a “wanting” response. The result is that when dopamine release is low, there can be a state of unhappiness characterized by endorphin deficiency. At the same time, general (usual) happiness depends on the dopamine homeostatic tonic set point.^6^ In addition to the coronavirus disease 2019 pandemic, there is a global addiction crisis. While being highest in the US, the devastation and deaths from drug overdose are global issues requiring “out of the box” thinking.^7^ Even in the face of harm reduction, relying on opioids to treat issues caused by other potent opioids seems counterintuitive and perpetuates unwanted addictions.^8^ Several investigative groups have been cognizant that addressing the root cause is one of the approaches to reducing harm.^9,10^ Another approach is using a narcotic antagonist (like naltrexone) to induce “psychological extinction” through blocking D2 receptors.^11^ The latter approach appears more acceptable; however, compliance remains a deterring issue.^12^ The approved drug acamprosate, an NMDA receptor antagonist and a positive allosteric modulator of GABAA receptors, also disrupts dopaminergic signaling.^13^ The growing acceptance of the RDS concept, introduced by Blum in 1995, facilitates the common mechanism hypothesis for substance and non-substance addiction. Understanding the in-common neuromodulating features of neurotransmission and its disruption through chronic exposure to substance and non-substance addictions requires the utilization of an approach that involves “dopamine homeostasis.”^14^

## 3. Review of evidence

The “out of the box” approach involves coupling genetic risk polymorphic testing with a safe and well-researched complex, KB220Z. The KB220Z is customized to match the presence of resultant alleles and provide a precision nutraceutical with known prodopamine regulatory pharmacological properties.^2,15^ High-tier publications strongly support a shared neuromechanism underlying both substance and non-substance addiction, such as alcohol, opioids, gambling, and food.

In the 1970s, Blum’s laboratory developed an amino-acid-based enkephalinase inhibitory pro-dopamine regulator with the KB220 nutraceutical complex as its cornerstone ingredient, now validated by over 45 clinical studies published in peer-reviewed journals.^16,17^ The basis of this complex is its ability to mimic the BRC,^17^ an established model of reward processing. The most striking feature is the activation of BOLD by the KB220Z across the BRC,^18^ including the NAc, anterior cingulate gyrus, anterior thalamic nuclei, hippocampus, prelimbic, and infralimbic parts of the prefrontal cortex (PFC). Evidence of genetic vulnerability as an antecedent to unwanted RDS behaviors may be a determining factor, which could be identified early in life. Based on previously published literature, the role of reward gene polymorphisms puts individuals at an increased risk for various forms of RDS behaviors, including anhedonia.^19,20^ This insight spurred the development of the patented genetic addiction risk severity (GARS) test, aimed at identifying genetic risk for these behaviors. Specifically, published studies have illustrated the coupling of GARS with KB220Z formulations of semi-customized precision pro-dopamine regulators tailored to one’s GARS profiles.^21^ The biological approach of this system enhances the effectiveness of RDS treatment.^22^

Balancing the BRC or achieving “dopamine homeostasis” is generally preferred and considered a commendable objective, as opposed to interventions that involve blocking natural dopamine or administering potent opioids to overcome opioid addiction.^21^ In the face of the current addiction pandemic, we urge addiction neuroscientists and clinicians to embrace this innovative technology and establish a “standard of care” for treating and preventing addiction and all related RDS neuro-sequala.^23^ While further research is required, it is crucial to establish a set of acceptable guidelines that include an understanding of the RDS concept. Understanding neurogenetics by utilizing a “systems biology” approach such as precision behavioral management, as outlined herein, seems prudent and represents a step forward in restoring well-being to the billions afflicted globally.^24–27^ In terms of a system biology approach, Rosen *et al*. outlined the theory behind complex trait analysis and systems genetics. They describe web-accessible resources, including GeneNetwork, that facilitate rapid exploratory analysis and hypothesis testing. Moreover, GeneNetwork is a tightly bioinformatic integrated tool and data set, allowing investigation into complex networks of gene variants, molecules, and cellular processes that modulate complex traits such as behavior and disease susceptibility. This technique will enable scientists to analyze gene expression across various specific brain regions and tissues, explore genetic covariance among traits, and map loci that modulate these traits. Rosen *et al*. further suggested that these tools enable investigators to assess the complex interactions of gene networks, employing a systems approach.^28^

## 4. Neurogenetic and epigenetic correlates of adolescent predisposition to and risk for addictive behaviors as a function of PFC dysregulation

Within the medical community, especially among addiction professionals, there is growing concern about how preteens, adolescents, and young adults turn to substance abuse to cope with stress and anger. The turbulence of the underdeveloped central nervous system (CNS), especially the PFC, underscores the need for continued neuroimaging studies in both human and animal models, as well as encourages preventive measures and regulatory actions taken by governmental bodies.

The PFC is known to undergo significant developmental changes before individuals reach their 20s, impacting decision-making ability within this population. Furthermore, early genetic testing for addiction risk alleles will provide valuable information that could potentially be utilized by parents and caregivers before any psychoactive drug use begins. Beyond genomic testing, a more straightforward approach could be the widespread adoption of a standard BHC, such as school fitness programs.

Family history, parenting styles, and relationship attachments, modified by various reward genes, including the well-known bonding substances oxytocin/vasopressin, may affect dopaminergic function. In addition, well-characterized neuroimaging studies indicate region-specific differential responses to drugs, food, and non-substance-addictive behaviors via either “surfeit” or “deficit.”^29,30^ Therefore, a “reward deficiency solution system” that combines early genetic risk assessment, medical monitoring, including a BHC, and nutrigenomic dopamine agonist modalities to combat reward deficiency risk may help address the global crisis that is hindering youth from leading normal, productive, and happier lives.^31^

Unlike fully developed adults, preteens transitioning into adolescence may lack adequate decision-making capacity due to incomplete brain development and myelination. The PFC area, known as the “braking/inhibitory system,” supports executive function and decision-making but can be hijacked by subcortical structures in the midbrain. Impairments in the midbrain region, which regulates social and emotional responses, may lead to deficits in neurotransmitter function.

We must be cognizant of the impact of stress on the brain’s developmental process and how substance abuse, such as alcohol, cocaine, and opioids, alters the integrity of white and gray matter volume.^32^ Furthermore, it is well known that myelination in the PFC begins when people are in their early 20s.^33–40^ Myelination regulates brain speed and can be compromised by stress and drug exposure, especially during prenatal and other developmental phases.^37–39^ During the turbulent years before adulthood, youth may encounter stressful situations, resulting in frustration that could trigger epigenetic changes that exacerbate genetic antecedent risk for drug abuse.^40,41^ The D2 dopamine receptor (*DRD2*) is the most extensively investigated gene in diverse neuropsychiatric disorders. Numerous international studies have been performed since the first association of the TaqI A DRD2 minor (A1) allele with severe alcoholism in 1990. As of October 10, 2022, there are 5351 articles listed in PUBMED, with 120 meta-analyses yielding mixed results. In our opinion, negative reports on the association of various *DRD2* gene polymorphisms are due to poorly screened controls, resulting in the non-elimination of many hidden RDS behaviors. Moreover, pleiotropic effects of *DRD2* variants have been observed in neurophysiologic, neuropsychologic, stress response, social stress defeat, maternal deprivation, and gambling disorders, whereby epigenetic DNA methylation and histone post-translational negative methylation have been identified in many citations.^14,42–56^ Methylation of *DRD2* has been observed in many facets of addiction, including increased striatal response to reward cues in alcoholics,^54^ decreased functional connectivity of the executive control network,^43^ and withdrawal.^44,46^ Blum and Noble characterized the *DRD2 Taq A1* allele as a generalized reward gene rather than one specific to alcoholism. This underscores the need for the field to find ways to either use effector moieties to edit the neuroepigenetic insults or possibly harness the idea of potentially removing negative mRNA-reduced expression by inducing “dopamine homeostasis.”

It is important to consider oxytocin as a crucial element in inducing dopamine balance within the brain. Evidence suggests an important interaction between oxytocin/vasopressin and dopamine function, as demonstrated by Modestino *et al*.^57^ This important interaction should not be ignored, especially in instances of antisocial behavior in youth, including those with conditions such as autism spectrum disorder.^58^

## 5. Opting for immediate satisfaction relative to delayed higher reward value in you

According to Volkow and Baler,^41^ it is imperative and critical for survival to learn how to balance behaviors that provide a reward NOW versus behaviors that provide an advantage LATER. Specifically, Volkow’s group proposed a model in which dopamine can favor NOW processes through phasic signaling in reward circuits or LATER processes through tonic signaling in control circuits. At the same time, through modulation of the orbitofrontal cortex, which processes salience attribution, dopamine enables shifting from NOW to LATER. In addition, modulation of the insula, which processes interoceptive information, influences the probability of selecting actions NOW versus LATER based on an individuals physiological state. Disruptions along these circuits contribute to diverse pathologies, including obesity, excessive reward-seeking behaviors, and various types of addiction.^59^

It is noteworthy that adolescents with a family history of substance use disorder (SUD) are at a greater risk for SUD. Rodriguez-Moreno *et al*.^60^ suggested that this may be partly attributed to the inheritance of behavioral impulsivity. They employed a delay discounting task to compare impulsivity in decision-making and its associated brain functioning among adolescents with and without a family history of substance abuse. During the task, subjects had to choose between “smaller, sooner” or “larger, later” rewards. The group with a family history of substance abuse displayed greater impatience by responding to “smaller, sooner” rewards more frequently compared to those without a family history of abuse. Behavioral impulsivity is ascribed to the differential developmental trajectories of two brain systems in young individuals. To provide clarity for those unfamiliar, it is known that children can be described with regard to how closely they are functioning to age-expected development in the three early childhood outcomes measured for federal reporting purposes. This is evaluated by collecting a variety of formative assessment data and using it to rate the child’s functioning on a 1 – 7 Likert scale, with 6 and 7 being the age-expected functioning level. In fact, the aim is to link performance with age expectation by comparing the functioning of children with disabilities to those developing according to age expectation. Specifically, Steinberg^61^ reported on the dominating role of the socioemotional brain systems in driving reward-seeking behavior in the face of an underdeveloped self-regulatory system. Casey’s group^62,63^ suggested that adolescent developmental changes are hierarchical in subcortical and cortical regions and their interconnections. For clarity, a hierarchy (from Greek: ἱεραρχία, *hierarkhia*, “rule of a high priest,” from *hierarkhes*, “president of sacred rites”) is an arrangement of items (objects, names, values, categories, etc.) represented as being “above,” “below,” or “at the same level as” one another.

Most importantly, it is plausible that in adolescence, over-activation of the brain’s reward system and under-activation of the cognitive control brain mechanisms can lead to unwanted substance-seeking behavior driven by impulsivity and sensation-seeking tendencies.^64^ Others suggested that choosing Now versus Later involves developmental changes that load onto poor decisions due in part to an undeveloped reward and cognitive control system, unlike their adult counterpart.^65–69^

## 6. Cognitive impairment in youth

In terms of cognitive impairment, especially concerning deficient executive cognitive functioning (ECF) in children, Aytaclar *et al*.^70^ reported that early adolescents at high risk for addictive behavior due to fathers with SUD demonstrated significantly poorer performance on ECF compared to lower risk adolescences. High-risk individuals in early adolescence displayed an earlier initiation of cannabis use and a greater prevalence of lifetime cannabis and tobacco use. Importantly, the level of ECF activity was predictive of the severity of drug involvement, including conduct problems and the number of drugs ever tried.

Several contributing factors are associated with cognitive impairment in youth, including but not limited to excessive opioid/alcohol intake in mothers during pregnancy,^71,72^ substance abuse, food addiction, and neuropsychiatric illnesses such as attention deficit hyperactivity disorder (ADHD) and attention deficit disorder.^73^ Bihlar Muld *et al*.^74^ highlighted that the clinical characteristics of patients with both ADHD and SUD differed from those with only SUD or ADHD and other psychiatric conditions, indicating the disabling nature of ADHD when combined with SUD. Specifically, the combination of severe substance abuse and ADHD resulted in poor general cognitive ability, including antisocial behavior. In addition, disruptions in the nascent synaptic networks and glia induced by opioids can impact brain connectivity and cognition after the opioid supply is abruptly stopped after birth.^75^ Neuroimaging has revealed abnormalities in brain structure, including cortical development, white matter microstructure, and functional connectivity, in newborns with fetal alcohol syndrome. These impairments in brain development modify developmental trajectories, leading to deficits in cognition, executive function, memory, behavior, and social adaptation.^72^ These catastrophic deficits in brain development pose risks for impending RDS behaviors, including SUD.

Undoubtedly, the prevalence of sugar in food and beverages has led to excessive consumption across all age groups, especially children and adolescents. It is staggering to note that over 60 countries consume sugar more than 4 times (>100 g/person/day), exceeding the World Health Organization’s (WHO) recommendations (25 g/person/day). Utilizing a validated mouse model, Beecher *et al*.^73^ reported that prolonged sugar overconsumption induces an abnormal response to novelty and changes both episodic and spatial memory. Their findings revealed that hippocampal-dependent learning and memory deficits accompany altered hippocampal neurogenesis. Specifically, there was an overall reduction in the proliferation and differentiation of neurons, especially within the dentate gyrus of newborns.

While the global obesity epidemic has been widely publicized in the media, understanding the evolution of sugar addiction could shed light on this dilemma. Avena’s group^76^ highlighted that the dopaminergic system in the mesolimbic region of the human brain is involved in hedonic rewards as a function of eating highly addictive, palatable foods like sugar. Particularly interesting is the role of acetylcholine in counteracting the dopaminergic surge as a plausible mechanistic action to help curb uncontrollable sugar cravings.

## 7. Proposing BHC as a novel program in the US’s educational system

In 2021, over 100,000 individuals died prematurely from an opioid overdose. Neuropsychiatric and cognitive impairments are underreported comorbidities of reward dysregulation due to genetic antecedents and epigenetic insults. Recent genome-wide association studies involving millions of subjects revealed frequent comorbidity with SUD in a sizeable meta-analysis of depression.^77^ Significant associations were identified between the expression of *NEGR1* in the hypothalamus and *DRD2* in the NAc, among other genetic factors. However, despite the rise in SUD and neuropsychiatric illness, especially in youth, routine standard objective assessments of brain function remain absent.

The importance of exercise programs in the global educational system was emphasized in 2020 by the release of updated global guidelines by the WHO on physical activity and sedentary behavior for children, adolescents, adults, older adults, sub-populations such as pregnant and postpartum women, and those living with chronic conditions or disabilities. According to Chaput *et al*.,^78^ increased and higher intensities of physical activity, as well as a diversity of physical activity (i.e., aerobic, muscle, and bone strengthening activities), are associated with improved health outcomes (primarily intermediate outcomes), as supported by various systematic reviews. Similarly, Thanos’s group^79^ reported that exercised rats had 18% and 21% lower dopamine D1R-like binding levels than sedentary rats within the olfactory tubercle and NAc shell, respectively. In addition, there was greater dopamine D2R-like binding in the NAc core (24%) and shell (25%) of exercised rats compared with sedentary rats. These observations support the hypothesis that aerobic exercise results in changes in the mesolimbic pathway that could mediate exercise-induced attenuation of drug-seeking behavior. The role of exercise, especially in the educational system, may have potential benefits for assisting school-age children with a positive family history of SUD, for example, through formal fitness programs.^80^

We propose that integrating existing education-based fitness programs with a standard BHC could synergistically not only improve the health of individuals but could also facilitate early identification of cognitive impairments. For early identification of cognitive abilities, DNA analysis through genetic testing, such as the GARS test, could provide important information, reflecting students’ brain neurotransmitter function at a genetic level.^19,21,78,80,81^

The rationale for encouraging a standard objective BHC is to acquire an extensive dataset to treat clinical syndromes in psychiatric patients and high-risk populations. While we advocate for implementing a generalized BHC across all K1–K12 students, its importance is especially pronounced for high-risk children attending “recovery high school (RHS).” Spearheaded by one of us (AJF) and others is the needed development of RHSs that provide a supportive educational and therapeutic environment for students following SUD treatment. According to Weimer *et al*.,^82^ most students served by RHSs have concurrent mental health disorders and are at risk for school failure, dropout, and substance use relapse. Fairly recently, RHS student high school graduation rates were 21 – 25 percentage points higher compared to students not attending RHS.^82^ This finding was statistically significant, albeit with limitations related to non-randomized design, selection bias in the study conditions, and uncertainty in calculating school costs. In another study by Tanner-Smith *et al*.,^83^ students attending RHS exhibited less frequent delinquent behavior while intoxicated and fewer days of substance use after discharge from SUD treatment than students attending non-RHS. Therefore, we propose RHS students as suitable candidates to test out the utilization of the BHC.

The proposed BHC comprises a set of reliable, accurate, and cost-effective objective assessments involving the following domains: (i) episodic and general memory; (ii) processing speed; (iii) attention; (iv) neuropsychiatry; and (iv) neurological imaging. After a review of over 36 years of computerized and written assessments primarily from PUBMED of memory, attention, psychiatric, and neurological imaging, the following recommendations have been selected for inclusion in the BHC: (i) MemTrax (episodic memory and processing speed); (ii) CNS vital signs (general and remote memory); (iii) test of variables of attention (attention); (iv) millon clinical multiaxial inventory III (neuropsychiatric); and (v) quantitative electroencephalogram/P300/evoked potential (neurological imaging). Continued research aims to simplify the BHC by including qEEG/P300/evoked potentials and genetically guided precision induction of “dopamine homeostasis.”^84^ This approach allows the assessment and treatment of reward deficiency and helps prevent dopamine dysregulation from being epigenetically transmitted to future generations.

During adolescence, developmental changes in the neural circuitry of reward processing, motivation, cognitive control, and stress may contribute to vulnerability to increased engagement in substance use and nonsubstance addictive behaviors.^85^ It has been suggested that the adolescent’s liability for addictions involves changes in the function and structure of the midbrain dopaminergic system, genetic antecedents, and epigenetic insults such as stress-induced neuroplasticity, contributing to imbalances between cognitive control and reward response.

Potenzas’ group^85^ suggests that leveraging genetics, epigenetics, and intermediate phenotypes/endophenotypes may help identify children and adolescents at risk. Once identified, it is crucial for these individuals to participate in a guidance program, essentially brain health coaching (BHCo). The advent of molecular neurobiological tools to uncover neurotransmitter cascade surfeits or deficits and possibilities for restoring dopamine balance across these brain regions, including the PFC, can improve screening of cognitive abilities, which would enhance prevention and intervention approaches. However, implementing changes in educational programs requires top-down public policy strategies. A detailed description of our proposed BHC can be found in Braverman *et al*.^86^

## 8. Epigenetics of reward processing in adolescence

It is widely acknowledged that the adolescent brain matures through a prolonged reorganization of gray matter, white matter, and associated neurochemical systems. Interestingly, this period of enhanced cognitive ability in adolescents coincides with a reduction in cortical gray matter thickness, resulting from epigenetic experience-dependent loss of synapses and a concomitant strengthening of the remaining connections.^87–89^ In addition, during adolescence, gray matter volume and density decrease in the brain, specifically in the parietal cortex, PFC, and basal ganglia, all of which are critical for executive function, motivated behaviors, and sensory processing.^34,90,91^ Furthermore, Paus^89^ demonstrated that there were corresponding increases in white matter, potentially reflecting augmented myelination and axonal diameter, leading to enhanced efficiency of impulse transduction. Notably, Gogtay *et al*.^92^ observed that phylogenetically older brain regions mature earlier than the newer ones. This delayed, uneven maturation of subcortical, emotional, and reward-focused systems, including cortical executive and impulse control systems, could underlie many RDS behaviors, including SUD.^93–95^

The prevalence of mental health disorders, including addictive behaviors, in children and adolescents has increased at least two- to three-fold from the 1990s to the present day.^87^ According to Monaco,^95^ one plausible mechanistic reason for this increase may be the transmission of altered brain circuits epigenetically across generations through non-DNA-based mechanisms (intergenerational and transgenerational effects). These epigenetic insults to the developing brain may be due to a family history of SUD, obesity, or a poor diet (e.g., processed, palatable foods). These insults may cause intergenerational and transgenerational effects for at least up to 2 years, influencing set points in neuropathways integrating sensory-motor, reward, and feeding behaviors.

In line with this, Hurd’s group linked parental THC exposure in rats to reduced proenkephalin mRNA expression in the NAc during early development, along with elevated expression during adulthood. Perinatal THC exposure also resulted in shorter latency to the first active lever press, greater responses to low heroin doses, and more heroin-seeking during mild stress and after extinction.^96^ Studies by Yuan *et al*.,^97^ and others^98^ reveal that persistent alterations in neuronal signaling and cognitive ability result from chronic nicotine exposure, likely due to altered dopamine function in the brain. Dopamine D_2_ receptor activation of fast-spiking interneurons in the PFC does not occur until late adolescence, along with the recruitment and maturation of local GABAergic activity.^99,100^ In addition, Tseng and O’Donnell^99^ point out that D_1_-NMDA receptor interactions in cortical pyramidal neurons that are necessary for mature cognitive and attentional processing continue to develop during this period. Flores-Barrera *et al*.^101^ discovered that ventral hippocampal input to the medial PFC is strengthened during late adolescence due to the D_1_ receptor-mediated emergence of NMDA receptor GluN2B subunit function. Unfortunately, in the mesolimbic system, particularly in the NAc, D_1_ and D_2_ receptor responses are immature, leading to reduced synaptic interaction between NAc and the PFC.^102^ Furthermore, the stimulation of the D_2_ receptor has an age-specific influence on AMPA-evoked cell excitability, and interactions between D_2_ and AMPA receptors elicit the activation of GABA interneurons, primarily in adults but not adolescents.^103^ In summary, these observations suggest a functional switch in reward processing during adolescent development mediated by dopamine regulation of GABA interneurons. It is well known that enhanced GABA transmission following chronic alcohol intake significantly reduces dopamine release at the NAc.^104^ In addition, stimulation of GABAB receptors inhibits dopaminergic VTA neurons.^105^ However, Pandey’s group demonstrated that the inhibition of VTA neuronal firing by bath‐applied GABA is primarily mediated by GABAA receptors.^106^

**Figure 2.**
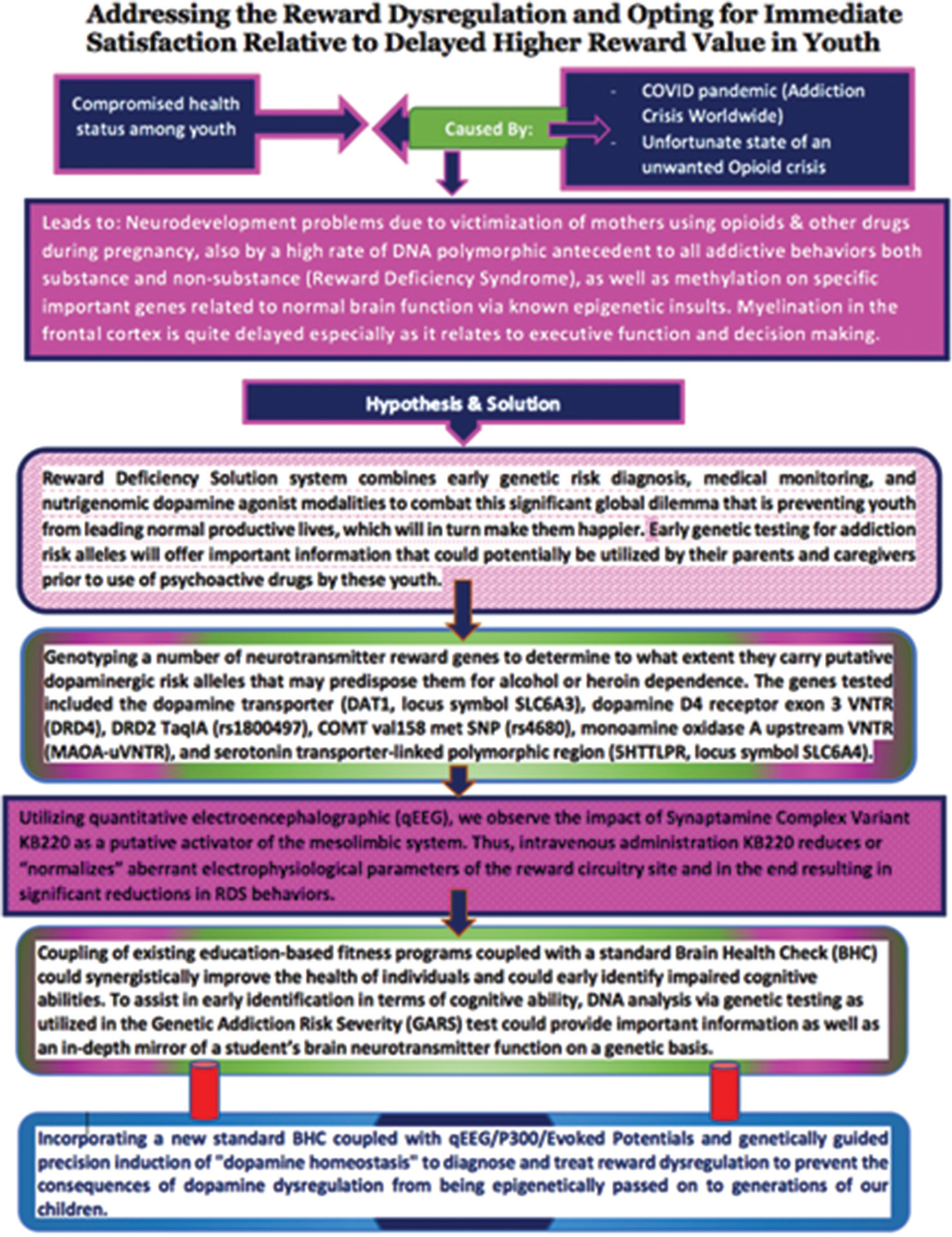
A conceptual schematic that summarizes reward dysregulation in youth and how the reward deficiency solution and brain health check can be used to diagnose and treat reward dysregulation.

The risk of all addictive drug and non-drug behaviors, especially in the unmyelinated PFC of adolescents, is both critical and complex. Many animal and human studies have highlighted the epigenetic impact on the developing brain in adolescents compared to adults. Some studies reveal an underlying hyperdopaminergia, which predisposes young individuals to risky behaviors by inducing high quanta presynaptic dopamine release at reward site neurons. In addition, altered reward gene expression in adolescents caused by epigenetically transferred social defeat, such as bullying, can persist into adulthood. However, there is also evidence that overstimulating epigenetic events can elicit adolescent hypodopaminergia. This complexity (Figure 2) suggests that neuroscience cannot definitively claim that all adolescents carry a hyperdopaminergic trait. To help dissect these seemingly opposing views, Blum’s laboratory reported a high risk for any addictive behavior (hypodopaminergia), especially drug-seeking (95%) and alcohol-seeking (64%) based on GARS testing of 24 Caucasians, ages 12–19 (derived from families with RDS). These results, although from a small cohort, should encourage further extensive studies in this area.

Mental disorders are widespread globally, influencing every community and age group, and contribute substantially to the overall disease burden, with major economic and social consequences as well as effects on human health and rights. Alarmingly, the largest inequities exist across nations, with 80% of people affected by mental disorders living in low- and middle-income countries, which benefit from scarcely 10% of global mental health resources. Unfortunately, poor rural areas in the US experience a significantly higher rate of mental disorders, including RDS behaviors such as SUD. Furthermore, due to low income and high juvenile delinquency in rural communities, possibly linked to cognitive inabilities such as poor decision-making, the recommendation of a standard BHC seems prudent. While globally accepted diagnostic categories and classifications, such as the Research Domain Criteria project, WHO International Statistical Classification of Diseases-11, or DSM-5, can help overcome global mental health challenges, our concern is that anomalous brain activity is not being adequately considered within the context of “systems biology,” neglecting educational, economic, and behavioral consequences that require appropriate and effective interventions. The best approach to achieving positive clinical outcomes is to initiate novel strategic alternative modalities targeting the etiology rather than just the symptoms.^107,108^

## 9. Positive thinking in adolescence

Positive emotions and cognition have been widely recognized for their beneficial effects on overall mental health and well-being, particularly when people focus on positive thought processes. In positive psychology, the goal is typically to engender character traits such as optimism and hope, which reduce anxiety and depression while fostering strong social interactions.^109^ There are many interventions aimed at developing and adjusting emotional and social skills in school, such as social and emotional learning programs^110^ or positive youth development interventions.^111^ However, research concerning positive thinking in adolescents has been relatively limited.^112^ Data indicate that negative emotions, such as anxiety or depression, are associated with the dysregulation of the amygdala – PFC circuitry.^113^ Positive emotional words are associated with increased activation in the ventral medial PFC.^114^ Other studies have uncovered important connections between positive emotions and brain processes relevant to prosocial behaviors. For example, a study of the positive emotion of professional pride revealed a relationship to empathy, reward, and emotion regulation, as well as the theory-of-mind network.^115^

From a neurotransmitter perspective, positive emotions are associated with increases in dopamine function within the reward network.^116–119^ Altered activity in serotonin modulates negative emotional responses.^120^ Oxytocin, which supports affiliative behaviors, may also play a role in responses to positive versus negative emotional processes.^121^ Thus, fostering positive emotions and implementing interventions that support them lead to substantial changes in the brain, involving various areas associated with reward, positive self-image, prosocial behaviors, and empathy. Working toward instilling positive emotions in adolescents is likely to yield short- and long-term benefits regarding their overall mental health and well-being.

## 10. Conclusion

Importantly, initial engagement in rehabilitation and detoxification bears similarities to experiencing a first stroke or heart attack in that the brain has already been impacted by pathological events that led to the manifestations of SUD and the need for treatment. The tools to prevent the progression of SUD are available and must be implemented urgently because deaths attributed to SUD have continued to increase unabated. Therefore, a reexamination of approaches to brain health and addiction and novel perspectives needs to be implemented by the medical community.

The clinical evidence accumulated during the past three decades underscores the necessity for establishing a BHC focused on precision neuropsychiatric testing, including episodic memory and processing speed (MemTrax),^122–124^ general memory (CNSVS),^125–130^ attention (T.O.V.A),^131–137^ neuropsychiatric (MCMI-III),^138–140^ and neurological imaging (qEEG/P300/EP),^131,137,141–154^ for patients at risk of or presenting with problematic drug misuse. Since addiction is related to learning mechanisms, refocusing on learning and memory may change the perspective on the beneficial use of these brain mechanisms. For example, the online program MemTrax (www.memtrax.com) can help individuals monitor their memory as frequently as needed and observe how it is being impacted by substance abuse. Such feedback can lead to behavioral improvements and serve as a valuable tool for those providing therapeutic interventions, such as BHCo.

One of the basic neurochemical mechanisms in the brain, the midbrain dopamine system, participates in pacing critical cognition functions, including reward and the facilitation of addictive behaviors.^155–158^ A study conducted by Rouhani and Niv,^155^ through fitting reinforcement learning models to behavior, demonstrated that both signed (cue predicting the reward) and unsigned (unexpected, surprising reward) prediction errors (RPEs) contribute to learning by modulating the learning rate. They further characterized the effects of these RPE signals on memory, demonstrating that both signed and unsigned RPEs augment memory, aligning with midbrain dopamine and locus-coeruleus modulation of hippocampal plasticity. Further research by this group supports the complex nature of reward and learning involving dopaminergic mechanisms.^156^ Finally, Katzman and Hartley’s work indicates that both children and adults tend to remember past events more when the value of choice is beneficial compared to non-beneficial.^157^ These proposed BHC/BHCo can be used as a standardized approach for school-aged children, akin to fitness programs. Our suggestion introduces a set of objective brain assessments parallel to those used in cardiology for diagnosing and following the clinical course of cardiac diseases. The coaching approach, including close evaluation and management guidance (including GARS testing and subsequent KB220z variant matching), could easily be adapted for implementation throughout the US and global educational systems. We understand that this initiative would require a substantial and bold approach to the care of the general US population. This commission believes that the BHC/BHCo would synergize with current fitness programs, particularly in addressing co-occurring RDS behaviors. Thanos *et al*.^158^ underscore the role of exercise in preventing the initiation of cocaine use in adolescence, suggesting that the implementation of exercise programs might be an important preventive measure and significantly improve students’ mental health. Based on the reviewed research, this appeal promises to stop/prevent the increased prevalence of SUD through early detection utilizing robust brain screening^159–209^ as recently proposed by the Society of Brain Mapping and Therapeutics,^84^ as well as psychological and pharmacological treatment approaches espoused herein.^210–257^

Notably, each year, over a million adolescents globally succumb to preventable or treatable causes. Psychosocial factors are the strongest factors associated with drug abuse, bullying, attempted suicide, and sleep deprivation resulting from bullying.^258–316^ The Carter Center has estimated that the addiction crisis, if continues to worsen at the same rate, may cost the US approximately 16 trillion dollars by 2030. Furthermore, the neurodevelopment of children could be compromised by maternal usage of opioids and other drugs during pregnancy. A high rate of DNA polymorphic antecedents compounds the epigenetic insults involving the methylation of specific essential genes related to normal brain function. Myelination in the frontal cortex, a process known to extend until the late 20s, delays proficient executive function and decision-making abilities. Understanding this delay in brain development, along with the presence of potential high-risk antecedent polymorphic variants or alleles and generational epigenetics, provides a clear rationale to mimic fitness programs with an adaptable BHC. Implementing the BHC within the educational systems in the US and other countries might be a good starting point for proactive therapies aimed at reducing juvenile mental health problems and, eventually, criminal activities, addiction, and other behaviors associated with RDS.
